# Study of self-esteem and perceived stress in medical students in Tunisia

**DOI:** 10.1192/j.eurpsy.2023.677

**Published:** 2023-07-19

**Authors:** M. Abdelkefi, R. Masmoudi, R. Jbir, S. Hentati, I. Feki, R. Sellami, J. Masmoudi

**Affiliations:** psychiatry A, Hedi Chaker university hospital, sfax, Tunisia

## Abstract

**Introduction:**

Self-esteem affects people’s reaction to stressful events and the way individuals cope with stress. At the same time, stressful events negatively affect self-esteem, which is a psychological resource against psychological disorders.

**Objectives:**

To study the link between self-esteem and perceived stress in medical students.

**Methods:**

A cross-sectional study was conducted through an online survey among medical students of the faculty of medicine of Sfax (Tunisia). Participants completed an anonymous self-administered questionnaire and two psychometric scales: Rosenberg’s self-esteem scale for the evaluation of self-esteem and Cohen’s Perceived Stress Scale (PSS) for the evaluation of the level of perceived stress.

**Results:**

Our sample consisted of 95 students. Their mean age was 25.8±3.4, with a sex ratio (M/F) = 0.25.The majority were single (83.2%) and live with their parents (64.2%). Only 14.7% of the participants were smokers, and 13.6% consumed alcohol.

History of psychiatric disorders was reported by 17.9% of students, 76.5% of which were anxiety disorders.

On the Rosenberg scale, self-esteem was very low in 27.1% and low in 34.7% of the students. According to the PSS scale, 21.1% had a severe stress level and 69.5% had a moderate stress level.

Female students had lower self-esteem with no significant correlation.

Students with a history of anxiety disorders had a significantly lower self-esteem (p<10^-3^).

Low self-esteem was significantly correlated with severe stress (p=0.01).

**Conclusions:**

Our study showed significant frequencies of low self-esteem and considerable stress among medical students. Low self-esteem was associated with severe stress. Further studies should be conducted to better investigate this relationship in order to promote student’s mental health and the use of stress management techniques, which can not only reduce stress, but also improve self-esteem.

**Disclosure of Interest:**

None Declared

